# Supplementary material for: Healthcare-seeking behaviours among mother’s having under-five children with severe wasting in Dodoma and Mbeya regions of Tanzania-A qualitative study
Source: PLOS Glob Public Health. 2024 Jan 8;4(1):e0001943. doi: 10.1371/journal.pgph.0001943 (PMC10773934; doi:10.1371/journal.pgph.0001943)
Supplement: S1 Appendix — (DOCX) [file pgph.0001943.s001.docx]

**S1 Appendix: FGD interview guide**

FOCUS GROUP DISCUSSION (FGD)

GUIDE FOR ALL MOTHERS/CAREGIVERS WITH CHILDREN LESS THAN 5 YEARS, MEN WITH CHILDREN LESS THAN FIVE YEARS AND INFLUENTIAL PEOPLE IN THE COMMUNITY

**Section 1: Identification**

Date: ______________________ Starting Time: _________ Ending Time: ___________

Region: ______________________________

District: ____________________________

Ward: _______________________________________

Name of Community/Village: ______________________________________________

Name of Facilitator: ________________________________

Name of Note Taker: ______________________________

Name of Observer: _______________________

***Instructions Moderator/interviewer: (please do not read out*)**

| *Before starting the interview make sure you have a consent form, tape recorder, notebook, pen and ink or stamp. You can phrase the questions/probes differently to suit the flow of the conversation*  *Do not need to ask every single probe, just make sure you cover the topic sufficiently before moving on.]* |
| --- |

**Step 1: Introduction:** Greet the participants and introduce yourself and the note-takers

**Step 2**: **Description of the study:** Provide a short description of the study objectives and explain why they have been selected to participate in the study and the duration of the interview.

**Step 3: Informed Consent**

Read through the consent form and ask them if they have got any questions or they need any clarification. For those who have agreed to participate the moderator will allow/assist them to sign the consent form. Provide ink or a stamp for participants who cannot sign.

**Step 4: Interview**

| **Characteristics of participants** | **P1** | **P2** | **P3** | **P4** | **P5** | **P5** | **P6** | **P7** | **P8** | **P9** | **P10** | **P11** | **P12** |
| --- | --- | --- | --- | --- | --- | --- | --- | --- | --- | --- | --- | --- | --- |
| Age |  |  |  |  |  |  |  |  |  |  |  |  |  |
| Sex |  |  |  |  |  |  |  |  |  |  |  |  |  |
| Marital status |  |  |  |  |  |  |  |  |  |  |  |  |  |
| Education level |  |  |  |  |  |  |  |  |  |  |  |  |  |
| Occupation |  |  |  |  |  |  |  |  |  |  |  |  |  |

***Marital status****: 1= Single, 2= Marriage / cohabited; 3= Divorced 4= Widow*

***Education****: 1= No school; 2=Primary; 3=Secondary; 4= Higher learning education*

***Sex****= 1=Male 2=Female*

***Occupation****= 1= Not working; farmer; 2=business 3= employed, 4=Others*

**Section 2: Knowledge of malnutrition**

1. What do you know about malnutrition?
2. What is the local name of malnutrition?
3. Can you tell how to do a child with malnutrition looks like (signs)?
4. What are the causes of malnutrition?
5. Do you think malnutrition is a problem in your community?

Probe: for the magnitude of malnutrition problem in the community? And which age group affected most?

1. Do you think that malnutrition can be treated? How & why

**Section 3: Health seeking behavior**

1. When children get sick(malnourished) what do caretakers/family normally do(Probe; foods, feeding practices, other local herbs given etc)
2. Who is normally consulted when a child is sick /malnourished in the family? Probes: Partner, Parents, in-laws, neighbors, Friends, influencers
3. Where is normally child taken for treatment/assistance? Probes; the H/F, Traditional healers, leaders? Pharmacy, church, mosque
4. If the any of above practices delays care/seek care early ask for reasons/motivation for doing that

**Section 4: Perception of the community on treatment of acute malnutrition**

1. What do your communities think of a child with malnutrition? Probe how do you think of the family with SAM children (being witched? Cursed)
2. In your community, how do you feel when you have a malnourished child? Probe: fear, rejection, stigma
3. Are there any beliefs related to malnutrition or care seeking? Probe: Traditions, foods cultures/norms, religious your child different food than you normally do?
4. Do you know the services available in the HF (name it) on malnutrition?
