# Supplementary material for: Healthcare-seeking behaviours among mother’s having under-five children with severe wasting in Dodoma and Mbeya regions of Tanzania-A qualitative study
Source: PLOS Glob Public Health. 2024 Jan 8;4(1):e0001943. doi: 10.1371/journal.pgph.0001943 (PMC10773934; doi:10.1371/journal.pgph.0001943)
Supplement: S2 Appendix — (DOCX) [file pgph.0001943.s002.docx]

**S2 Appendix: KII GUIDE**

KEY INFORMANT INTERVIEW (KII)

GUIDE FOR ALL MOTHERS/CAREGIVERS WITH CHILDREN LESS THAN 5 YEARS, MEN WITH CHILDREN LESS THAN FIVE YEARS AND INFLUENTIAL PEOPLE IN THE COMMUNITY.

**Section 1: Identification**

Date: ______________________ Starting Time: _________ Ending Time: ___________

Region: ______________________________

District: ____________________________

Ward: _______________________________________

Name of Community/Village: ______________________________________________

Name of Facilitator: ________________________________

Name of Note Taker: ______________________________

Name of Observer: _______________________

**Information for interviewee**

Age ________________________________________

Gender__________________________________________

Maritial status______________________________________

Education level______________________

Occupation ________________________

**Section 2: Understanding on malnutrition**

1. What do you know about malnutrition?
2. What are the causes of malnutrition?
3. Do you think malnutrition is a problem in your community?

-Probe: for the magnitude of malnutrition problem in the community?

-And which age group affected most?

1. Do you think that malnutrition can be treated? How & why
2. Do you think Malnutrition can be prevented and how?

**Section 3: Health seeking behavior**

*(Start by identifying her/his feelings if she/he has a malnourished child now or had a malnourished child in the past)*

1. How did you identify your child when she got malnutrition?
2. What was the first things/aid did you do at home (Probe for food/drink given, local herbs)
3. When your child got sick/ malnourished who did you consult? **Probe**: Partner, Parents, in-laws, neighbors, Friends, influencers
4. Did you take your child for treatment? Probes; where, Traditional healers, religious leader /church Pharmacy?
5. If the any of above practices delays care/seek care early ask for reasons/motivation for doing that

**Section 4: Perception of community of acute malnutrition &services**

1. What does your community think of a child with malnutrition? Probe how do you think of the family with a SAM children (being witched? Cursed)
2. In your community, how do you feel when you have a malnourished child?

***Probe:*** fear, rejection, stigma

1. How do people in the community take care of a child with malnutrition?
   1. Probe: Should you give your child different food than you normally do?
   2. Do you know the services available in Health centers/dispensaries on malnutrition?

**Section 5: Factors that influence accessibility of acute malnutrition**

1. What factors prevent people from sending children to the hospital early when they have symptoms of malnutrition?

Probe; not know the services available to Health center, the quality of the services, Distance, economic activities, waiting for long time , Stigma, fear, decision making.
