# Supplementary material for: Healthcare-seeking behaviours among mother’s having under-five children with severe wasting in Dodoma and Mbeya regions of Tanzania-A qualitative study
Source: PLOS Glob Public Health. 2024 Jan 8;4(1):e0001943. doi: 10.1371/journal.pgph.0001943 (PMC10773934; doi:10.1371/journal.pgph.0001943)
Supplement: S3 Appendix — (DOCX) [file pgph.0001943.s003.docx]

**S3 Appendix: Codebook**

| **Code name** | **Descriptions** |
| --- | --- |
| 1. **Awareness and risk factors on acute malnutrition** | Apply this code when a participant describes the   - meaning of acute malnutrition - Signs and symptoms - Causes of the problem, - Magnitude of malnutrition and reasons for such magnitude. - Perceived magnitude of malnutrition and reasons for such magnitude. - Risk factors for SAM? |
| 1. **Community perception about malnourished children** | - Use this code when a participant describes insights about how people in the community perceive a malnourished child (cursed, bewitched, neglected, stigmatized etc) - Perceived causes of malnourishment |
| **Community/parent/caregiver’s action on malnourished children/help seeking patterns/pathways**   1. **Care giver’s and community response on malnourished child** | Use this code when a participant mentions   - The first place they go for malnutrition treatment, - reasons for preferring those places, - The reasons for preferring health facilities (if mentioned health facility as the first place) or reasons for preferring going elsewhere mentioned.   Use this code when a participant explains about   - Feeding practices for malnourished children and - Types of foods given to their malnourished children.   What members of community or CHW do in the event of malnourished child in community |
| 1. **Parent/caregiver’s malnutrition treatments experience** | Apply this code when a participant describes   - treatment service(s) to their malnourished children received and the ways they have been treated at the health facility, or elsewhere - Any other health service given by CHW, - All explanation whether satisfied with services offered. - Perception of quality of services received - Perception of costs if any - Health providers attitude/reactions during treatment |
| 1. **Barriers for early healthcare seeking among community members** | - Use this code to label all reasons that have been mentioned and described by a participant to delay healthcare seeking behaviors among parents/caregivers. - Note both community level barriers and health systems barriers |
| 1. **Facilitators for early healthcare seeking among community members** | - Use this code to describe all reasons that have been explained by a participant to motivate healthcare seeking behaviors among parents/caregivers on treatments of malnutrition. |
| 1. **Reasons for defaulting malnutrition treatments** | - Apply this code when a participant explains about things that make a parent/caregiver not to complete malnutrition treatment as advised by healthcare professionals. |
| 1. **Ways for improving healthcare seeking behavior** | - Use this code when a respondent describes the strategies, they have been using to facilitate healthcare seeking behaviors among parents/caregivers of the under-fives with acute malnutrition. |
| 1. **Recommendations for improving healthcare seeking behavior** | - Apply this code when a participant suggests ways/methods to improve healthcare seeking behaviors among parents/caregivers of the malnourished children. |
| 1. **Emerging issues** | - Use this code when a participant mentions anything important but not covered on the previous codes. |
