# Supplementary material for: Healthcare-seeking behaviours among mother’s having under-five children with severe wasting in Dodoma and Mbeya regions of Tanzania-A qualitative study
Source: PLOS Glob Public Health. 2024 Jan 8;4(1):e0001943. doi: 10.1371/journal.pgph.0001943 (PMC10773934; doi:10.1371/journal.pgph.0001943)
Supplement: Attachment — Submitted filename: A rebuttal letter_10_10_2023.docx [file pgph.0001943.s005.docx]

Dear Razak M Gyasi, PhD, PD

Thank you very much for giving us the opportunity to submit a revised draft of our manuscript titled “Mother’s healthcare-seeking behaviours in Dodoma and Mbeya regions of Tanzania- A qualitative study of children with acute malnutrition”
We appreciate the time and effort that you and reviewers have dedicated to providing valuable feedback on our manuscript.

Here is a point-by-point response to the reviewers’ comments and concerns.

**Review Comments to the Author**

**Reviewer #1**

**Comment 1**: Firstly, I would to appreciate the researchers for raising cross cutting nutrition concerns that will improve or change the current trend of treatment and prevention of severe acute malnutrition.
**Response:** Thank you very much and, we really appreciate your insightful comments and suggestions.

**Comment 2:** Researches used the term acute malnutrition and severe acute malnutrition interchangeable including the research title which is not appropriate. I suggest contextualization of the idea including the research tile “Mother’s healthcare-seeking behaviours in Dodoma and Mbeya regions of Tanzania- A qualitative study of children with severe acute malnutrition”.

**Response:** Thank you very much and we agree with your comment. We have re-write the terms ‘acute malnutrition’ and ‘severe acute malnutrition’ to ‘severe wasting’ as suggested by the WHO Note on Terminology of “Wasting” and “Acute malnutrition” of February 2023.

**Comment 3**: How did you come-up with 96 study participants? Which formula did you used? Justify?

**Response:** Thank you very much and, we have added information and a reference that justifies our sample of 96 participants (see page 6, paragraph 2).

**Reviewer #2
Comment 1:** Title:

The title of the study is confusing. The words “Children with acute malnutrition" following qualitative study is misleading

**Response:** Thank you very much and we agree with your comment. We have rephrased the title.

**Comment 2**: Abstract:
2a.In methodology part of abstract, thematic analysis which was mentioned in the main body of manuscript have to be included along with content analysis.

**Response**: Thank you very much for pointing out this and, we have added thematic analysis in the methodology part of abstract.

**Comment 3**:

2b. Distance of health facility would be rather considered a health system factor than environmental factor

**Response**: We agreed with this suggestion and rephrased the sentence as suggested.

**Comment 4**: Methods
4a: A qualitative study employs various research methods which include Ethnography, phenomenology, Grounded theory and case studies. The authors have to mention which research method was done rather than using cross- sectional study

**Response:** Thank you very much and we agreed with this comment and added the Ethnography method.

**Comment 5**:

4b: With respect to study setting, authors can simply state the regions and districts were selected based on case load using purposive sampling.

**Response**: We agree with your comment and rephrased the paragraph.

**Comment 6**: 5) Results
5a: Results section also to mention regarding various themes generated from the transcripts.

**Response:** Thank you very much and we have mention ‘various themes generated from the transcripts’ in the result section.

**Comment 7**: 6) Conclusion
6a: Can be limited to one paragraph

**Response:** We agreed with comments and limited the conclusion to one paragraph.

Sincerely,

Ray Masumo, PhD
