# Supplementary material for: Healthcare-seeking behaviours among mother’s having under-five children with severe wasting in Dodoma and Mbeya regions of Tanzania-A qualitative study
Source: PLOS Glob Public Health. 2024 Jan 8;4(1):e0001943. doi: 10.1371/journal.pgph.0001943 (PMC10773934; doi:10.1371/journal.pgph.0001943)
Supplement: Attachment — Submitted filename: A rebuttal letter2_14_11_2023.docx [file pgph.0001943.s006.docx]

Dear Razak M Gyasi, PhD, PD,

Once again, thank you very much for giving us the opportunity to submit a second revised draft of our manuscript titled “Healthcare-seeking behaviours among mother’s having under-five children with severe wasting in Dodoma and Mbeya regions of Tanzania- A qualitative study ” to *PLOS Global Public Health*.

We appreciate the time and effort that you and reviewers have dedicated to providing valuable feedback on our manuscript.

Here is a point-by-point response to the journal requirement and reviewers’ comments and concerns.

**Journal Requirement**

**Comment 1:** *Please review your reference list to ensure that it is complete and correct. If you have cited papers that have been retracted, please include the rationale for doing so in the manuscript text, or remove these references and replace them with relevant current references. Any changes to the reference list should be mentioned in the rebuttal letter that accompanies your revised manuscript. If you need to cite a retracted article, indicate the article’s retracted status in the References list and also include a citation and full reference for the retraction notice.*

**Response**: We highly appreciate this comment and, we have carefully reviewed the reference list, and ensured it is complete and correct.

**Comment 2:** *We have noticed that you have uploaded Supporting Information files, but you have not included a list of legends. Please add a full list of legends for your Supporting Information files after the references list.*

**Response:** Thank you very much for pointing this out and, legends for supporting information files have been added.

**Review Comments to the Author**

**Reviewer #1** **Abdirahman Ahmed**

**Comment 1:** *Manuscript is acceptable for publication. In point-point respond my name is not mentioned. Try to add.*

**Response:** Please accept our sincere apologies. We highly accept your insightful comments and suggestions.

**Reviewer #2**

**Comment 1:** *I would like to thank the authors for submitting revised manuscript. However, I have noticed that only the title has been changed. The authors, need to provide explanation for the rest of the comments.*

**Response:** Thank you very much for pointing this out and all the requested changes have been incorporated into the manuscript (abstract, introduction, methodology, results and discussion, conclusion).

Sincerely,

Ray Masumo, PhD
